# Geographic disparities and socio-demographic predictors of pertussis risk in Florida

**DOI:** 10.7717/peerj.11902

**Published:** 2021-08-31

**Authors:** Corinne B. Tandy, Agricola Odoi

**Affiliations:** Biomedical and Diagnostic Sciences, University of Tennessee, Knoxville, Tennessee, United States

**Keywords:** Pertussis, Tango’s spatial scan statistics, Geographic information systems, GIS, Cluster detection, Ordinary least squares regression, FlexScan, Flexible scan statistics

## Abstract

**Background:**

Pertussis is a toxin-mediated respiratory illness caused by *Bordetella pertussis* that can result in severe complications and death, particularly in infants. Between 2008 and 2011, children less than 3 months old accounted for 83% of the pertussis deaths in the United States. Understanding the geographic disparities in the distribution of pertussis risk and identifying high risk geographic areas is necessary for guiding resource allocation and public health control strategies. Therefore, this study investigated geographic disparities and temporal changes in pertussis risk in Florida from 2010 to 2018. It also investigated socioeconomic and demographic predictors of the identified disparities.

**Methods:**

Pertussis data covering the time period 2010–2018 were obtained from Florida HealthCHARTS web interface. Spatial patterns and temporal changes in geographic distribution of pertussis risk were assessed using county-level choropleth maps for the time periods 2010–2012, 2013–2015, 2016–2018 and 2010–2018. Tango’s flexible spatial scan statistics were used to identify high-risk spatial clusters which were displayed in maps. Ordinary least squares (OLS) regression was used to identify significant predictors of county-level risk. Residuals of the OLS model were assessed for model assumptions including spatial autocorrelation.

**Results:**

County-level pertussis risk varied from 0 to 116.31 cases per 100,000 people during the study period. A total of 11 significant (*p* < 0.05) spatial clusters were identified with risk ratios ranging from 1.5 to 5.8. Geographic distribution remained relatively consistent over time with areas of high risk persisting in the western panhandle, northeastern coast, and along the western coast. Although county level pertussis risks generally increased from 2010–2012 to 2013–2015, risk tended to be lower during the 2016–2018 time period. Significant predictors of county-level pertussis risk were rurality, percentage of females, and median income. Counties with high pertussis risk tended to be rural (*p* = 0.021), those with high median incomes (*p* = 0.039), and those with high percentages of females (*p* < 0.001).

**Conclusion:**

There is evidence that geographic disparities exist and have persisted over time in Florida. This study highlights the application and importance of Geographic Information Systems (GIS) technology and spatial statistical/epidemiological tools in identifying areas of highest disease risk so as to guide resource allocation to reduce health disparities and improve health for all.

## Background

Pertussis is a toxin-mediated respiratory illness caused by *Bordetella pertussis* that can result in severe complications, particularly in infants less than year old (CDC, 2015). Severe complications include pneumonia and neurological complications. Between 2008 and 2011, children less than 3 months old accounted for 83% of pertussis deaths in the United States. While most of the cases and especially those that are severe occur in infants, people of all ages are susceptible to the disease (Clark, 2014) and adolescents and adults are considered the most important sources of infection for susceptible infants (CDC, 2015; Esposito & Principi, 2016).

Prior to the development of a vaccine, pertussis was a universal disease of childhood and infancy. Immunization programs initiated in the 1950s dramatically reduced the incidence of the disease in the United States until the 1980s, when a re-emergence occurred (Chiappini et al., 2013). While the cause(s) of the re-emergence are still unclear (Clark, 2014), the severity of disease in infants makes ensuring high vaccination coverage in all age groups a public health necessity. The rise in cases of the disease among vaccinated adolescents and school-aged (5–9 years) children (Clark, 2014) has resulted in concerns that the efficacy and length of the period of immunity provided by the vaccine may not be as long as initially thought (Jackson & Rohani, 2014; Schwartz et al., 2016; Esposito et al., 2019). Although vaccines have been well-established as one of the safest and most successful tools to control and prevent infectious diseases, doubt of vaccine efficacy as well as concerns over side effects has increased and has resulted in decreasing vaccination coverage (Hickler et al., 2015). Research indicates that variations in vaccination coverage as well as socioeconomic and demographic factors has contributed to changes in pertussis epidemiology (Omer et al., 2008; Atwell et al., 2013; Clark, 2014). Vaccine hesitancy has been shown to be influenced by demographic, socioeconomic, and attitudinal factors. For example, minority populations often have lower vaccination rates (Williams et al., 2015). Additionally, socioeconomic status and level of education are major predictors of vaccination acceptance (Blue & Valley, 2002; Larson et al., 2014). Since pertussis is a vaccine-preventable disease and demographic and socioeconomic factors influence acceptance and uptake of vaccines, it is important to investigate and identify how these factors impact pertussis vaccination and disease epidemiology.

A number of studies have reported temporal trends in the occurrence of pertussis (Broutin et al., 2010; Bouchez & Guiso, 2015; Cherry, 2015). Epidemics of the disease have been reported to occur approximately every 3–5 years across the globe (Broutin et al., 2010; Clark, 2014; CDC, 2015). This cyclical pattern is seen in the United States as well (Broutin et al., 2010; Bouchez & Guiso, 2015). Bouchez and Guiso have suggested that these cycles result from an increase in susceptible hosts which is influenced by demographic differences and varying vaccine coverage (Bouchez & Guiso, 2015). They also suggest that temporal changes in disease surveillance may play an important role in the observed cyclical nature of pertussis epidemics (Bouchez & Guiso, 2015).

Between 2000 and 2008, the average annual incidence of pertussis in the United States was 3.4 per 100,000 and between 2009 and 2016, the average incidence rose to 5.9 per 100,000 (Skoff, Hadler & Hariri, 2019). An investigation of the geographic distribution of pertussis in the United States from 2000 to 2016 (Skoff, Hadler & Hariri, 2019) showed that pertussis incidence was highest in the central mid-west (*i.e*. Kansas, Nebraska, South Dakota) [11.9 per 100,000] and Rocky Mountain (*i.e*. Colorado, Montana, Utah) [11.5 per 100,000] areas. Lowest annual average incidence was seen in the south Atlantic (*i.e*. Delaware, Georgia, North Carolina) [2.5 per 100,000] and southeastern (*i.e*. Tennessee, Kentucky, Alabama) [3.1 per 100,000] areas.

The state of Florida has seen a rise in pertussis cases and, as of 2010, it was the only vaccine-preventable disease with consistent case increases in that state (Schulte et al., 2010). As of 2019, Florida had a pertussis incidence of 1.85 per 100,000 (Centers for Disease Control & Prevention (CDC), 2019). In a study of pertussis in Florida, Schulte et al. (2010) reported that the incidence increased from 0.44 per 100,000 in 2000 to 1.7 in 2006. This increase in incidence over time mirrors trends reported across the United States. The authors also assessed geographic patterns and identified a cluster of ten counties that reported 66% of the cases seen in the state (Schulte et al., 2010).

Geographic Information System (GIS) analyses of health data have become increasingly important in the investigation and control of both chronic and infectious diseases. Spatial epidemiologic methods, ranging from descriptive disease maps to visualize spatial disparities in disease distribution to advanced spatial models, are critical in understanding geographic disparities in disease risk and their determinants. Findings from such investigations help identify populations at highest risk and provide useful information to guide health planning, resource allocation, disease control programs and policy. Thus, findings of this approach can often lead to more targeted, cost-effective intervention and prevention efforts. Kauhl et al. (2017), in their analysis of pertussis testing and incidence in the Netherlands, suggested that GIS methods and geographically weighted regression be considered for identifying pertussis outbreaks and to better direct disease control strategies. Additionally, a study by Aloe, Kulldorff & Bloom (2017) investigated nonmedical vaccination exemptions and pertussis outbreaks in the United States using GIS. They demonstrated the utility of using geospatial analysis to explore sociopolitical predictors of vaccine-preventable diseases such as pertussis and reported that high rates of exemptions can compromise herd immunity at small geographic scales (Aloe, Kulldorff & Bloom, 2017).

Investigating the spatial epidemiology of pertussis is useful in identifying: (a) geographic disparities of the disease and (b) determinants of the identified disparities. Moreover, identification of the predictors of high pertussis risk is important in guiding resource allocation so as to target control programs to reduce disease risk and improve population health (Siegal et al., 1997). Therefore, this study investigated geographic disparities and temporal changes in pertussis risk in Florida from 2010 to 2018. It also investigated and identified socioeconomic and demographic predictors of the identified geographic disparities in pertussis risk in Florida.

## Methods

### Study area

**Figure 1:**
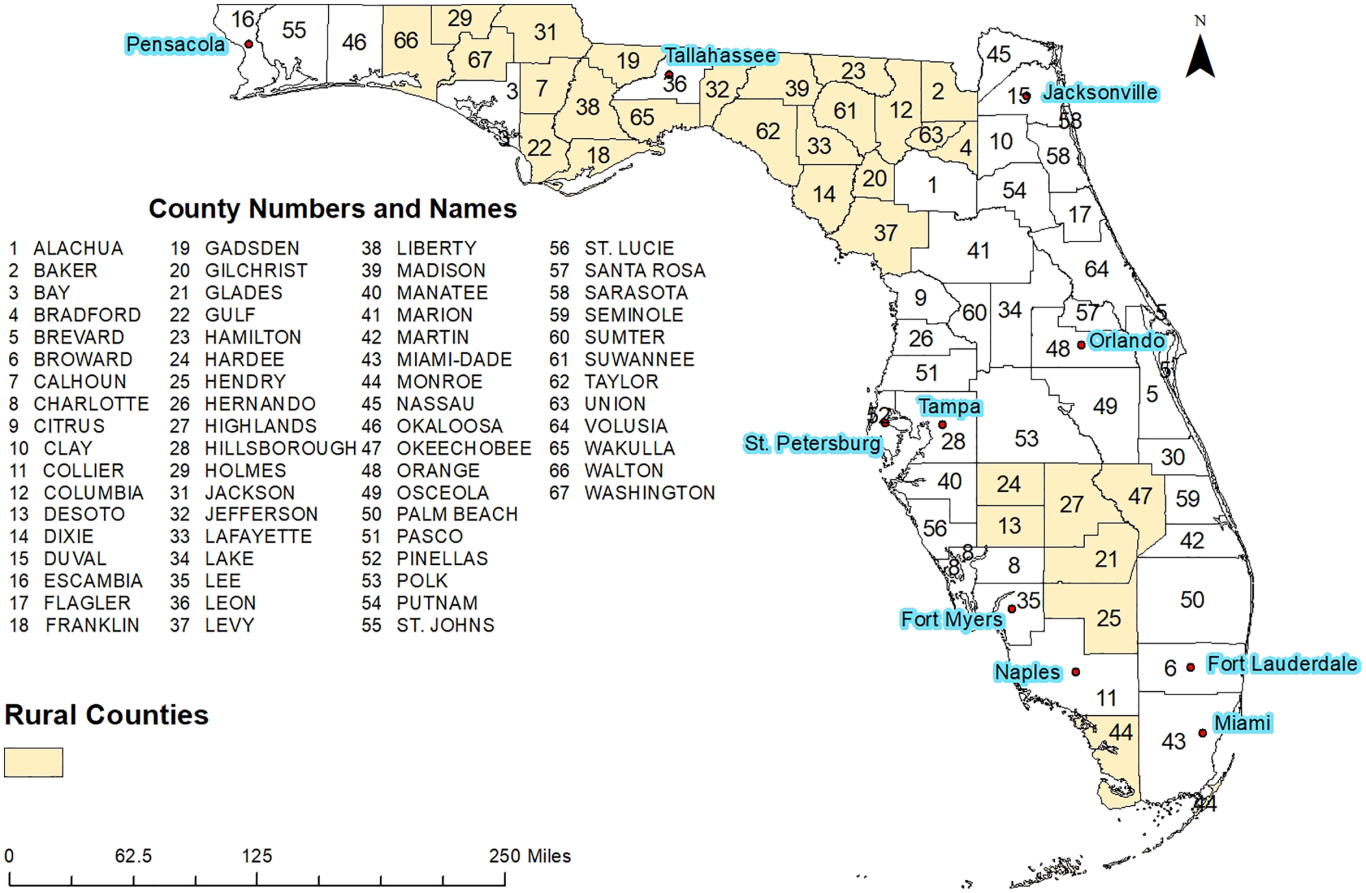
Geographic distribution of major cities, rural and urban counties of Florida.

This retrospective study investigated pertussis risk in the state of Florida, which consists of 67 counties, both rural and urban (Fig. 1). Miami-Dade County is the most urban and most populated with approximately 2.7 million residents, while Liberty County is the most rural and least populated county with approximately 8,300 residents (US Census Bureau, 2018). Florida is 75.4% White, 25% Hispanic, and 16.1% Black or African American (US Census Bureau, 2018). The state is 51.1% female and 48.9% male and has the following age distribution: <5 years (5.4%), 5–9 years (5.5%), 10–14 years (5.7%), 15–19 years (5.8%), 20–24 years (6.2%), 25–34 years (12.9%), 35–44 years (12.1%), 45–54 years (13.3%), 55–59 years (6.8%), 60–64 years (6.4%), 65–74 years (10.9%), 75–84 years (6.2%), and ≥85 years and older (2.6%) (US Census Bureau, 2018).

### Data sources and variable selection

Yearly number of cases of pertussis for each county for the time period 2010–2018 were obtained from the Florida Department of Health. Cases were aggregated into three time periods: 2010–2012, 2013–2015, and 2016–2018. The 3-year aggregation was necessary due to the very small numbers of yearly cases observed and the fact that pertussis occurrence has a 3–5 year periodicity (Broutin et al., 2010; Clark, 2014; Bouchez & Guiso, 2015; CDC, 2015). Population data were downloaded from Florida Health CHARTS, a community health assessment tool created by the Florida Department of Health (Florida Department of Health, 2019). Vaccination exemption data were also obtained from the Florida Department of Health. All cartographic boundary files used for creating maps were downloaded from the United States Census Bureau’s TIGER files and the State of Florida Geographic Data Portal (Southwest Florida Water Management District, 2019).

County level age-adjusted pertussis risks were computed and presented as number of cases per 100,000 population. A total of 20 potential predictors of pertussis risk were identified and considered for investigation at the county level: education, sex, race/ethnicity, income level, employment, health insurance, number of primary care physicians, percentage of population living in rural areas, vaccination coverage, and vaccination exemptions (Table 1).

**Table 1:** Variables considered as potential predictors of county-level pertussis risk in Florida.

Variable theme	Specific variable
Education	% Less than High School
	% Completed High School
	% Completed Bachelor’s Degree
Race and Ethnicity	% White, Non-Hispanic
	% Black, Non-Hispanic
	% Hispanic
Sex	% Male
	% Female
Economic	% Unemployed
	% Family Living Below Poverty
	% Individual Living Below Poverty Median Income
Vaccination	% Vaccinated in K-12 Population
	% Total Exemptions % Temporary Medical Exemptions % Permanent Medical Exemptions % Religious Exemptions
Healthcare	% Uninsured
	Primary Care Physicians per 100,000 population
Geography	Rural County (Yes or No)
	% Population Living in a Rural Area

### Investigation of high-risk clusters of pertussis

Tango’s flexible spatial scan statistic (FSSS) was used to identify the geographic locations of high-risk clusters of pertussis using FleXScan (Tango & Takahashi, 2005). Since the flexible spatial scanning window detects both circular and irregularly shaped clusters (Jacquez, 2009; Odoi et al., 2019; Lord, Roberson & Odoi, 2021), it is ideal for situations like this one involving investigation of clusters whose shapes are unknown at the outset (Roberson et al., 2019). It has been shown that Tango’s flexible scan statistics has good power as well as the ability to detect irregularly shaped clusters more accurately than Kulldorff’s spatial scan statistics (Tango & Takahashi, 2005). Poisson probability model was used specifying maximum spatial scanning window size of 15 counties. Clusters were identified based on likelihood ratio tests. For statistical inference, 999 Monte Carlo replications were used, and the null hypothesis of total spatial randomness was rejected if the *p*-value was ≤0.05.

### Investigation of predictors of county-level pertussis risk

Predictors of county-level pertussis risk were investigated using an ordinary least squares regression model. The first step in building the model involved fitting univariable models between each of the potential predictors and the outcome variable (log-transformed age-adjusted pertussis risk). Univariable associations were assessed at a liberal alpha of 0.2 and therefore variables with a *p* < 0.2 were considered for further investigation in step two. Two-way correlations were run for variables considered for step 2. Only one of a pair of highly correlated variables (absolute value of *r* > 0.7) were assessed in step two. The decision regarding which of a pair of highly correlated variables to include in step two was based on biological and statistical considerations. The second step involved fitting multivariable ordinary least squares model using manual backwards elimination with the predictor variables identified for inclusion in step one. During this step statistical significance was assessed using an alpha of 0.05. Confounding was investigated by assessing whether the removal of a variable resulted in a >20% change in the coefficients of any other variables in the model (Dohoo, Martin & Stryhn, 2012). If a confounder was identified, it was forced into the model regardless of its statistical relationship with the outcome variable. Robust Lagrange Multiplier (RLM) tests, implemented in GeoDa, were used to assess for spatial autocorrelation in the residuals using queen spatial weight (Anselin, Syabri & Kho, 2016). Multicollinearity was assessed using both Variance Inflation Factor (VIF) in SPSS (IBM SPSS Statistics for Windows, Version 25.0; IBM, Armonk, NY, USA) and the Multicollinearity Condition Number in GeoDa (Anselin, Syabri & Kho, 2006).

### Cartographic displays

All geographic information system (GIS) manipulations and cartographic displays were performed in ArcGIS (ArcGIS Desktop 10.6.1. Esri, Redlands, CA, USA). Statistically significant county-level predictors of pertussis risks, age-adjusted pertussis risks, and significant clusters of pertussis risk were displayed in maps. The choropleth maps of pertussis risk and of high-risk clusters were generated for the full study period and three-year time periods: 2010–2012, 2013–2015, and 2016–2018. Critical intervals in maps of the determinants of pertussis risk as well as maps of pertussis risk were determined using Jenk’s optimization classification scheme (Jenks, 1967) for the full study period (2010–2018) and were applied to pertussis risk maps of other time periods for consistency.

### Ethical statement

This study was assessed by the University of Tennessee Institutional Review Board (IRB Number: UTK IRB-20-05957) which determined that it did not involve human subjects as defined in 45 CFR 46.102 (e) (1), since it did not involve use of identifiable private information. Thus, the IRB determined that neither IRB review, nor certification of exemption from review, was required.

## Results

### Geographic and temporal distribution of pertussis risk

**Figure 2:**
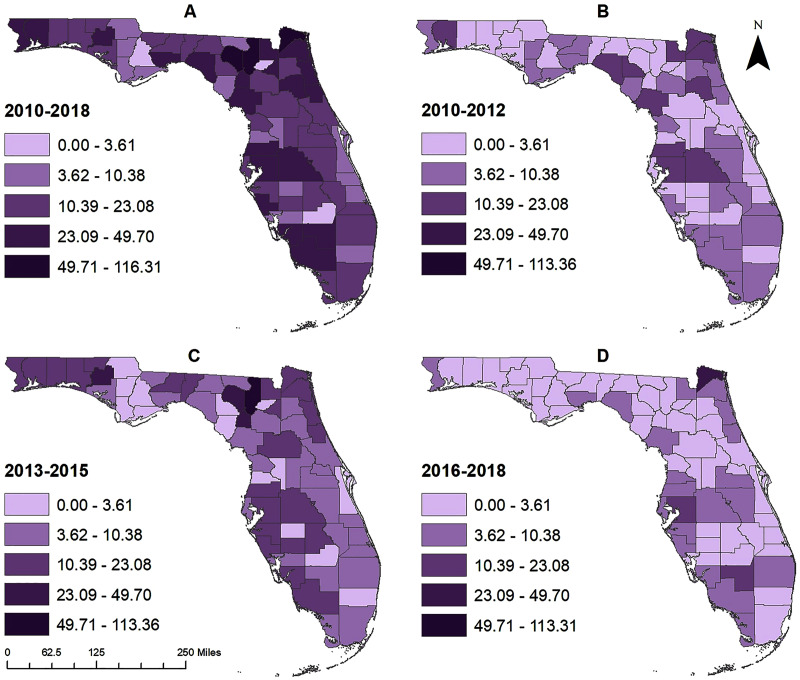
County-level geographic distribution of pertussis risk in Florida, 2010–2018. (A) 2010–2018, (B) 2010–2012, (C) 2013–2015, (D) 2016–2018.

County-level pertussis risk varied from 0 to 116.31 cases per 100,000 people during the study period (2010–2018) (Fig. 2A). Four regions with high risks of pertussis were evident: the Pensacola metropolitan area in the western panhandle (Escambia and Santa Rosa counties), the Tampa Bay area (Hillsborough, Pasco, and Polk counties), Jacksonville metropolitan area (Nassau, Duval, and St. Johns counties), and the Cape Coral-Fort Meyers-Naples Area (Collier, Hendry, and Lee counties) (Figs. 1 and 2A). High risks were also observed in Columbia and Suwannee counties. Areas of low risk, on the other hand, were evident around Glades, Union, Liberty, and Calhoun Counties (Figs. 1 and 2A).

The pattern of geographic distribution of pertussis risk was slightly different for the time period 2010–2012, compared to the whole time period (2010–2018), with high risks being observed in Santa Rosa county in the western Panhandle, around the northern gulf coast in Taylor, Lafayette, and Levy counties, in the northwestern region around Jacksonville in Nassau, Baker, Duval, and Putnam counties, and around the Tampa Bay area in Pasco, Hillsborough, and Polk counties (Figs. 1 and 2B). Though the general spatial patterns of pertussis risk for the time period 2013–2015 were similar to those of 2010–2012, a number of counties (such as Columbia, Suwannee, Washington, *etc*.) had higher overall risks in 2013–2015 than 2010–2012 (Figs. 1 and 2B–2C). In addition, high risks were identified in Gilchrist County, around the Tampa Bay area in Pasco, Hillsborough, Polk, Highlands, DeSoto, Manatee, and Sarasota counties, and around the Cape Coral area in Lee and Collier counties. Although county level pertussis risks generally increased from 2010–2012 to 2013–2015, risk tended to be lower during the 2016–2018 time period compared to the 2013–2015 period (Figs. 2C and 2D). Highest risks were observed in Nassau County followed by Nassau, Hillsborough and Hendry counties.

### Clusters of high pertussis risk

**Figure 3:**
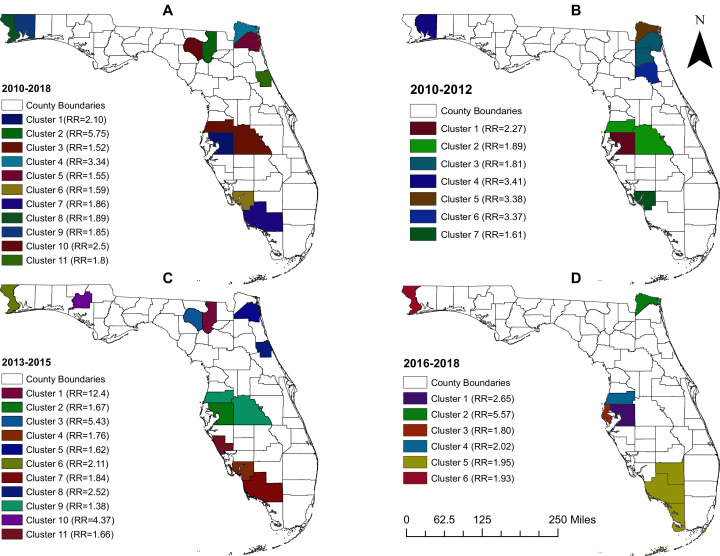
Geographic distribution of high-risk clusters of pertussis in Florida, 2010–2018. (A) 2010–2018, (B) 2010–2012, (C) 2013–2015, (D) 2016–2018.

A total of 11 clusters were identified for the analysis that included data for the whole study period (2010–2018) (Table 2 and Fig. 3A). The primary cluster during this time period included one county (Hillsborough) and had a risk ratio (RR) of 2.1. The risk of pertussis in this cluster was 43.1 cases per 100,000 and it had a total of 556 observed cases of pertussis when only 264 cases were expected. Based on geographic size, the largest cluster was Cluster 3 which included two counties (Polk and Pasco). The risk of pertussis in this cluster was 1.51 times higher than the risk outside the cluster. A total of 340 pertussis cases were observed in this cluster when only 223 cases were expected. It is worth mentioning that the cluster with the highest RR (Cluster 2) included only one county (Columbia) and had a RR = 5.7, a risk of 116.3 cases per 100,000 persons, 79 observed cases while only 13 were expected. Counties in the Tampa Bay area (Hillsborough, Polk, and Pasco counties) were consistently included in high-risk clusters throughout the study period (Fig. 3A).

**Table 2:** Significant geographic clusters of pertussis risk in Florida, 2010–2018.

Cluster	Population		Observed number of cases	Expected number of cases	Risk per 100,000 people	Risk ratio	*p*-value
1		1,307,906	556	264.45	43.10	2.10	0.001
2		67,924	79	13.7	116.3	5.75	0.001
3		1,106,654	340	223.7	32.28	1.52	0.001
4		75,569	51	15.2	67.48	3.34	0.001
5		893,858	281	180.7	31.88	1.55	0.001
6		656,466	212	132.7	32.44	1.59	0.001
7		338,270	127	68.3	37.54	1.86	0.001
8		304,654	117	61.6	38.40	1.89	0.001
9		160,475	60	32.4	38.01	1.85	0.001
10		44,263	22	8.9	49.70	2.46	0.01
11		99,646	37	20.1	37.13	1.83	0.029

Seven clusters, having RRs ranging from 1.6 (in cluster 7) to 3.4 (in cluster 4), were identified during the time period 2010–2012 (Table 3 and Fig. 3B). The time period 2013–2015, on the other hand, had a total of 11 clusters with RRs ranging from 1.4 (in cluster 9) to 12.4 (in the primary cluster) (Table 4 and Fig. 3C). The latter was located in Columbia County and had the highest RR across all time periods. Collier county, in the Naples metropolitan area, and Nassau county, in the greater Jacksonville area, were also identified as parts of high-risk clusters during the time periods 2013–2015 (Fig. 3C) and 2016–2018 (Fig. 3D). The fewest number of clusters were identified in the 2016-2018 time period (Table 5 and Fig. 3D). This was also the only time period during which a multi-county high pertussis risk cluster (RR = 1.95; *p* = 0.002) was identified.

**Table 3:** Significant geographic clusters of pertussis risk in Florida, 2010–2012.

Cluster	Population	Observed number of cases	Expected number of cases	Risk per 100,000 people	Risk ratio	*p*-value
1	1,242,491	181	79.69	14.57	2.27	0.001
2	1,072,014	130	68.76	12.14	1.89	0.001
3	1,056,763	123	67.7	10.97	1.81	0.001
4	155,225	34	9.95	21.9	3.41	0.001
5	73,671	16	4.72	21.72	3.38	0.001
6	73,864	16	4.73	21.66	3.37	0.001
7	628,356	65	40.30	10.34	1.61	0.012

**Table 4:** Significant geographic clusters of pertussis risk in Florida, 2013–2015.

Cluster	Population	Observed number of cases	Expected number of cases	Risk per 100,000 people	Risk ratio	*p*-value
1	67,924	77	6.2	113.36	12.4	0.001
2	1,307,906	200	119.5	15.29	1.67	0.001
3	44,263	22	4.04	49.70	5.43	0.001
4	656,466	106	59.9	16.14	1.76	0.001
5	893,858	133	81.6	14.87	1.62	0.001
6	304,654	59	27.8	19.36	2.11	0.001
7	338,270	57	30.9	16.85	1.84	0.002
8	99,646	23	9.1	23.08	2.52	0.005
9	1,106,654	140	101.1	12.68	1.38	0.005
10	24,076	10	2.2	40.01	4.37	0.005
11	388,037	59	35.4	15.20	1.66	0.006

**Table 5:** Significant geographic clusters of pertussis risk in Florida, 2016–2018.

Cluster	Population	Observed number of cases	Expected number of cases	Risk per 100,000 people	Risk ratio	*p*-value
1	1,388,111	183	68.8	13.18	2.65	0.001
2	79,592	22	3.94	27.64	5.57	0.001
3	961,253	86	47.6	8.94	1.80	0.001
4	507,081	51	25.1	10.05	2.02	0.001
5	474,481	46	23.5	9.47	1.95	0.002
6	312,811	30	15.5	9.59	1.93	0.034

### Predictors of pertussis risk

Results of descriptive analysis of the potential predictors and assessment of their associations with pertussis risk are presented in Table 6. The following variables were assessed in the multivariable model: rural status, education variables, percent female, primary care providers per 100,000 population, median income, percent vaccinated, percent with medical exemption, and percent with religious exemptions. Only three of these variables were significant in the final model: rurality, county level median income and percentage of females in the county. County level pertussis risks tended to be high in rural counties (*p* = 0.021), those with higher median income (*p* = 0.039), and those with higher percentages of females (*p* < 0.001). It is worth pointing out that % female was a distorter variable for the association between rurality and risk of pertussis with the coefficient of the rural variable switching from a negative association with log-risk of pertussis, in the univariable model, to a positive association in the final model when percent females is added to the model (Tables 6 and 7). There was no evidence of multicollinearity as all the VIFs were <4 and the multicollinearity condition number was <20 (Table 7). Additionally, there was no evidence of non-normality of the residuals (Jarque-Bera test *p* = 0.678). Both the Robust Lagrange Multiplier tests for lag (LM_lag_ *p* = 0.585) and error (LM_error_ *p* = 0.472) showed no evidence of spatial dependence of the residuals.

**Table 6:** Summary statistics and results of univariable analysis of potential predictors of pertussis risk in Florida.

Variable	Median	1st Quartile	3rd Quartile	Unadjusted parameter estimate	Lower 95% CI	Upper 95% CI	*p*-value
Less Than High School Education	14.5	11.5	21.7	−0.313	−0.080	−0.011	0.010
High School Education	34.3	28.9	37.5	−0.258	−0.090	−0.003	0.035
College Education	18.6	11.4	26.7	0.269	0.003	0.056	0.028
White, non-Hispanic	83.0	77.8	87.6	0.094	−0.016	0.035	0.449
Black, non-Hispanic	13.1	8.6	19.1	−0.116	−0.038	0.014	0.351
Hispanic	8.65	5.29	18.1	0.040	−0.017	0.023	0.751
Male	49.4	48.6	53.3	−0.494	−0.184	−0.072	0.00
Female	50.6	46.7	51.4	0.494	0.072	0.184	0.00
Unemployed	6.6	5.8	7.2	−0.133	−0.334	0.099	0.282
Uninsured	18.1	15.6	21.1	−0.224	−0.106	0.004	0.069
Family Below Poverty Line	12.8	10.1	16.9	−0.164	−0.087	0.017	0.184
Individuals Below Poverty Line	17.7	14.3	22.5	−0.145	−0.075	0.019	0.24
Primary Care Providers (per 100,000)	48.0	28.0	68.0	0.305	0.003	0.021	0.012
% Living in Rural Area	23.8	8.5	67.5	−0.256	−0.015	−0.001	0.036
Rural County (Yes or No)	–	–	–	−0.254	−0.978	−0.028	0.038
Median Income	43063	36907	48483	0.307	0.095	0.719	0.011
% K-7^th^ Grade Vaccinated	92.9	89.9	95.1	−0.240	−0.108	0.000	0.051
% K-7^th^ Grade with Vaccination Exemptions	7.1	4.9	10.1	0.240	0.000	0.108	0.051
% K-7^th^ Grade with Medical Exemptions	1.7	0.8	2.3	0.138	−0.084	0.300	0.265
% K-7^th^ Grade with Religious Exemptions	2.4	1.2	3.5	0.178	−0.033	0.214	0.15

**Table 7:** Significant predictors of county-level pertussis risk in Florida, 2010–2018.

Variables	Exponentiated parameter estimate	Lower CI (95%)	Upper CI (95%)	*p*-value	VIF
Rural (vs. Urban)	1.511	1.134	4.540	0.021	2.82
Scaled Median Income ($10,000)	1.317	1.019	2.031	0.039	1.57
Percent Female	1.982	1.102	1.293	<0.001	2.17

### Geographic distribution of significant predictors of pertussis risk

**Figure 4:**
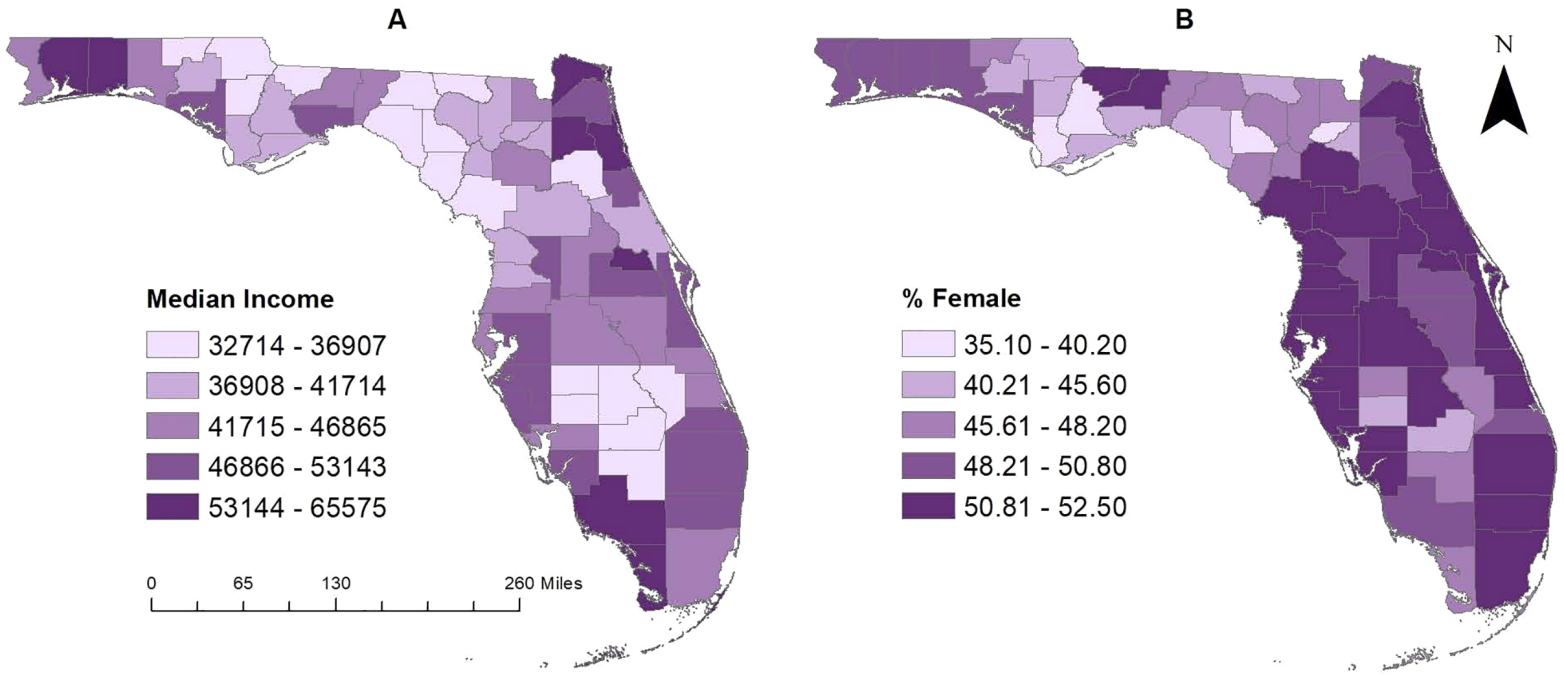
(A–B) County-level geographic distribution of significant predictors of pertussis risk in Florida, 2010–2018.

Geographic distributions of the three significant predictors of county-level pertussis risk are shown in Figs. 1 and 4. Unsurprisingly, the general distribution of these predictors is similar to the geographic distribution of pertussis risk in Florida between 2010 and 2018 (Fig. 2A). Santa Rosa and Okaloosa counties in the western panhandle, Nassau, St. John’s, and Clay counties in the Northwestern coastal area, Hillsborough, Manatee, and Sarasota counties in the Tampa Bay area, and Collier and Monroe counties in the Fort Myers area all had relatively high median incomes (Figs. 1 and 4A). These counties were also consistently identified as counties with high pertussis risk and in high risk clusters (Figs. 2A, 3A and 4A).

Counties with higher percentages of females are evident in the central panhandle (Gadsden and Hernando counties) and along the eastern and western coastal borders. Counties with greater than 52% female population included: Gadsden, Leon, Sarasota, Hernando, and Pinellas counties (Figs. 1 and 4B). All of these counties had high pertussis risk (>8 per 100,000) and many counties with higher female populations were identified in high risk clusters, including Hillsborough, Lee, Escambia, Santa Rosa, Duval, St. John’s, Polk, and Pasco counties. Finally, rural counties, were predominately located in the central and eastern panhandle and the central body of the state (Fig. 1).

## Discussion

This study investigated county-level geographic disparities and temporal changes in, as well as socioeconomic and demographic predictors of, pertussis risk in Florida from 2010 to 2018. Study findings provide information that is useful for guiding resource allocation for disease control programs. There is a dearth of research investigating the geospatial epidemiology of pertussis in the United States and spatial epidemiologic studies of pertussis predominantly explore the uptake of pertussis vaccines. While prevention of pertussis through education campaigns and vaccination efforts is critical, identifying the sociodemographic and economic predictors of pertussis risk cannot be overlooked. Therefore, it is necessary to investigate geographic disparities in risk of the condition and identify socioeconomic and demographic predictors of any identified geographic disparities in pertussis risk.

The results of this study indicate that geographic disparities of pertussis risk exist at the county level in Florida. This is consistent with findings from other studies that have reported spatial disparities in pertussis risk in the US (Siegal et al., 1997; Iroh Tam et al., 2016) and other countries (Kauhl et al., 2017; Mohammadbeigi et al., 2020). A study in Colorado analyzed data for the time period 1986–1994 and identified consistent clusters of high pertussis risk in the state (Siegal et al., 1997). The authors reported that high rates of pertussis were associated with census tracts with higher proportions of residents living below the poverty line (Siegal et al., 1997). An Iranian study of vaccine-preventable diseases at the district-level using data for the time period 2015–2018, reported that pertussis cases were identified in urban areas exclusively (Mohammadbeigi et al., 2020).

Findings from this study indicate that the risk of pertussis was higher during the time period 2013–2015 than any other time within the study period and a greater number of counties had higher risks during this time period. Temporal changes and epidemic cycles of pertussis are well documented in the literature (Fine & Clarkson, 1982; Broutin et al., 2010; Bouchez & Guiso, 2015). Broutin et al. (2010) conducted a comparative study of 64 countries and reported that pertussis epidemics were characterized by a 3–5 year cyclical pattern. They suggested that these temporal variations were due to regional variations of demographics, vaccination coverage, surveillance strategies, and changes in the circulating bacteria subtypes (Broutin et al., 2010; Bouchez & Guiso, 2015).

The current study also identified high-risk clusters of county-level pertussis risk around some major metropolitan areas in Florida. These findings are consistent with findings from other studies, including an analysis of pertussis in Ohio, which reported geographic clusters of pertussis cases around metropolitan areas (Rowe, 2006). Additionally, a comparative study of two outbreaks in Minnesota reported an association between population density and pertussis case density (Wi et al., 2019) which is consistent with high risk clusters being identified in large metropolitan areas. Similarly, an ecological analysis of pertussis in Minnesota reported that urban counties had 1.79 times higher risk of the disease than rural counties (Iroh Tam et al., 2016). Moreover, population density was also reported as a significant predictive factor, increasing pertussis risk by 6% for every 100 persons/km^2^ increase in population density at the public health unit-level in southern Ontario, Canada (Elghamudi & Berke, 2020). This is expected given that pertussis is transmitted through respiratory droplets, and hence has high transmissibility in crowded environments. Given the relatively high reproductive number of 15–17 of pertussis, it stands to reason that the more densely populated an area is, the more effective transmission will be (European Centre for Disease Prevention & Control, 2012).

### Predictors of pertussis risk

Significant predictors of county-level pertussis risk identified in the current study were percentage of females, median income, and rurality. Counties with high proportions of females tended to have high pertussis risk. This is consistent with findings from individual level studies that reported higher risks of pertussis among females than males in the United States, Canada, and Poland (De Serres et al., 2000; Paradowska-Stankiewicz & Rudowska, 2015) . An analysis of an outbreak of pertussis in Quebec, Canada, reported that the main risk factors for pertussis were being female and working in an educational or healthcare setting. The authors proposed that the higher risk may be attributed to greater awareness of pertussis among females which could lead them to seek medical care more readily than their male counterparts (De Serres et al., 2000). Higher pertussis risk and pertussis complications among females have also been reported in the United Kingdom and Canada (Jenkinson, 1995; Abu-Raya et al., 2020). Since the current study is not an individual-level study, the findings of the above individual-level studies cannot be directly implied to it. However, it is possible that the associations seen at the individual-level may apply at the county-level as well. Suffice it to say that this is the first ecological study that has investigated sex as a potential sociodemographic predictor of pertussis risk despite evidence at the individual level that sex is an important predictor of pertussis risk (Broutin et al., 2004; Huang et al., 2017; Alimohamadi et al., 2020).

The association between higher median income and higher county-level pertussis risk may be related to vaccine refusal since higher median income has been well-documented as associated with vaccine refusal for ideological reasons (Wei et al., 2009; Remes et al., 2014). However, it is worth noting that vaccination delay has also been identified as a significant contributor to disparities in vaccination coverage and often occurs along socioeconomic and demographic lines. Children from economically disadvantaged families, with poor access to healthcare services, often have delays in their vaccination schedules. This is of particular interest because this delay can increase risk of not only pertussis illness but other vaccine-preventable diseases as well (Luman et al., 2005; Feemster et al., 2009; Schaller, Schulkind & Shapiro, 2019). However, children with vaccine delays are usually fully vaccinated by the time they begin attending school since it is required unless an exemption has been approved (Schaller, Schulkind & Shapiro, 2019). This may explain why the current study did not identify an association between vaccination coverage and county-level pertussis risk, since we could not assess the vaccination coverage of children prior to starting compulsory kindergarten education.

While high-risk clusters were identified around metropolitan areas, the final multivariable model indicated that risk of pertussis was higher in rural counties than urban counties. These contradictory findings may be the result of distortion effects of percent females in the model. However, our finding that rurality is a predictor of higher county-level pertussis risk is consistent with reports of a geospatial analysis of pertussis in Saskatchewan, Canada, that rural areas had higher pertussis risk than non-rural areas (Medu et al., 2018). There is a clear need to explore the relationship between population density, rurality, and risk of pertussis more deeply, as there is conflict not only in this study but also in the literature. Possible explanations for these findings include lower vaccination rates due to lack of access to healthcare services or geographical differences related to objections to vaccinations.

### Strengths and limitations

To our knowledge, this is the first study to investigate spatial patterns and clusters of pertussis in Florida using rigorous spatial epidemiologic methods. The FSSS has several strengths, over other methods such as the Kulldorff’s spatial scan statistics and local indicators of spatial associations (LISA) methods such as Moran’s I: (a) it does not have the problem of multiple comparisons associated with LISA; (b) does not have pre-selection bias; (c) it is able to detect irregularly shaped clusters since it identifies all cluster shapes as opposed to Kulldorff’s spatial scan statistics which only identifies circular or elliptical clusters (Tango & Takahashi, 2005). Moreover, the Tango’s FSSS has been shown to work better than Kulldorff’s scan statistic for identifying small clusters (Tango & Takahashi, 2005). County level spatial analysis was necessary to avoid the small number problem associated with analysis at lower geographical scales because of the small numbers of cases of pertussis. The identified spatial clusters and predictors are useful for guiding health planning and policy. In addition to identifying county-level predictors of pertussis risk, this study has demonstrated the usefulness of using spatial scan statistic to identify areas where preventive resources are needed through the identification of high-risk clusters of pertussis. However, this study is not without limitations. Pertussis is an under-reported disease and, therefore, the county-level pertussis risk reported in this study is likely lower than the true risk. Due to the small number of cases and to protect anonymity and confidentiality of cases, only yearly pertussis data were used. These data were further aggregated into 3-year time periods for descriptive investigation to ensure stable estimates due to the small yearly case counts. Future studies will need to investigate individual level data covering longer study periods. These limitations notwithstanding, the findings of this study are important for guiding and planning public health programs.

## Conclusions

There is evidence that geographic disparities exist and have persisted over time in Florida. This study highlights the importance and application of GIS technology and spatial statistical/epidemiological tools in identifying areas of highest disease risk so as to guide targeting of resource allocation to reduce health disparities and improve health for all.

## Supplemental Information

10.7717/peerj.11902/supp-1 Supplemental Information 1 Study dataset.

## Additional Information and Declarations

### Data Availability

The following information was supplied regarding data availability: The raw data are available in the Supplemental File.
